# Overweight/obesity in adolescents with type 1 diabetes belonging to an admixed population. A Brazilian multicenter study

**DOI:** 10.1186/s13098-021-00759-9

**Published:** 2022-01-04

**Authors:** Marilia Brito Gomes, Deborah Conte, Karla Rezende Guerra Drummond, Felipe Mallmann, André Araújo Pinheiro, Franz Schubert Lopes Leal, Paulo Henrique Morales, Carlos Antonio Negrato

**Affiliations:** 1 Department of Internal Medicine, Diabetes Unit, State University of Rio de Janeiro, Rio de Janeiro, Brazil; 2 Department of Ophthalmology, Federal University of São Paulo, São Paulo, Brazil; 3 Department of Ophthalmology, Federal University of Rio Grande Do Sul (UFRGS), Rio Grande do Sul, Brazil; 4 Department of Ophthalmology, Regional Hospital of Taguatinga, Brasília, Brazil; 5 Department of Ophthalmology, University of Campinas, Campinas, São Paulo Brazil; 6 Medical Doctor Program, University of São Paulo- School of Dentistry, Rua Saint Martin 27-07, Bauru, São Paulo Zip Code: 17012433 Brazil

**Keywords:** Type 1 diabetes, Adolescents, Overweight, Obesity, Diabetes-related chronic complications, Glycemic control, Cardiovascular risk factors

## Abstract

**Background:**

To determine the prevalence of overweight/obesity and associated risk factors in Brazilian adolescents with type 1 diabetes (T1D) and its association with diabetic retinopathy (DR) and chronic kidney disease (CKD).

**Methods:**

This study was performed in 14 Brazilian public clinics in ten cities, with 1,760 patients. 367 were adolescents (20.9%):184 females (50.1%), 176 (48.0%) Caucasians, aged 16.4 ± 1.9 years, age at diagnosis 8.9 ± 4.3 years, diabetes duration 8.1 ± 4.3 years, school attendance 10.9 ± 2.5 years and HbA1c 9.6 ± 2.4%.

**Results:**

95 (25.9%) patients presented overweight/obesity, mostly females. These patients were older, had longer diabetes duration, higher levels of total and LDL-cholesterol, higher prevalence of family history of hypertension, hypertension, undesirable levels of LDL-cholesterol, and metabolic syndrome compared to eutrophic patients. No difference was found regarding ethnicity, HbA1c, uric acid, laboratorial markers of non-alcoholic fatty liver disease (alanine aminotransferase, aspartate aminotransferase, gamma-glutamyl transferase).

**Conclusions:**

Almost one quarter of our patients presented overweight/obesity. These patients had higher prevalence of traditional risk factors for micro and macrovascular diabetes-related chronic complications such as diabetes duration, hypertension, high levels of LDL-cholesterol and metabolic syndrome. The majority of the patients with or without overweight/obesity presented inadequate glycemic control which is also an important risk factor for micro and macrovascular diabetes-related chronic complications. No association was found between overweight/obesity with diabetic CKD, DR and laboratorial markers of non-alcoholic fatty liver disease. The above-mentioned data point out that further prospective studies are urgently needed to establish the clinical prognosis of these young patients.

## Background

Type 1 diabetes (T1D) is a common endocrine disorder found in adolescents worldwide, caused by an autoimmune destruction of pancreatic beta-cells [1]. For a long time, T1D was associated with a lean phenotype [1] but in the last decades, obesity has been present among these patients even at diagnosis [2]; however, it can also appear after the initiation of insulin treatment that can contribute to weight gain and clinical characteristics of insulin resistance [3]

Patients with T1D that present obesity and other clinical features of insulin resistance at diagnosis or those who gain weight during treatment are termed as having double-diabetes [4–6]. In general patients with overweight/obesity show other components of insulin resistance or metabolic syndrome (MS) that are risk factors for the presence of diabetes-related chronic complications (DRCC) [7–9]. A recent meta-analysis has found that approximately one quarter (23.7%) of patients with T1D were affected by MS [10]. The presence of MS or insulin resistance are considered as risk factors for the presence of poor glycemic control, CKD and cardiovascular diseases (CVD) [7] and retinopathy [9]. Recently, MS was also associated with the presence non-alcoholic fatty liver disease (NAFDL) in patients with T1D as has recently been demonstrated in a study carried out in a tertiary care center in our country [8]. In this study, patients with altered hepatic images on ultrasound or transient elastography had higher body mass index (BMI) and presence of MS [8].

Some studies have found an association between weight gain and intensive insulin therapy even in those using insulin pumps [6, 11, 12]. Subgroup analyses of the Diabetes Control and Complications Trial (DCCT) and the Epidemiology of Diabetes Interventions and Complications (EDIC) have found that patients who gained weight during the trial presented features associated with increased cardiovascular risk and those in the highest quartile for weight gain exhibited higher blood pressure and a more atherogenic lipid profile [6]. In the same study, those patients in the intensive insulin therapy gained twice as much weight compared to those under conventional care [11]. Greater weight gain was associated with poorer glycemic control at baseline, greater decrease in HbA1c levels, presence of severe hypoglycemic episodes but had no relationship with caloric intake and with physical activity intensity. It is supposed that a decrease in glycosuria and consequent better calorie utilization or even other unknown mechanisms are involved in this process [11, 13].

This study aims primarily to investigate the prevalence of overweight and/or obesity and its associated cardiovascular risk factors in Brazilian adolescents with T1D and secondly its association with DRCC.

## Methods

### Study design and data collection

This study had a cross-sectional design and was conducted in 10 Brazilian cities, from all geographic regions of the country, with patients followed in 14 public clinics between 2011/2014.

All patients received free health care (NPH and regular insulins, syringes, needles, glucometers and strips for blood glucose monitoring) from the Brazilian National Health Care System (BNHCS) Each clinic provided data for at least 50 T1D outpatients that were treated by an endocrinologist in secondary or tertiary care settings. Included patients were those with the diagnosis of T1D done by a physician, the need for continuous insulin use since the diagnosis, at least 13 years of age, and followed at each diabetes center for at least 6 months. Pregnant or lactating women, patients who had an acute infection or ketoacidosis in the three preceding months or had a history of renal transplantation were excluded [14].

The total sample was composed of 1760 patients. They were diagnosed as having T1D between 1960 and 2014. 367 patients (20.9%) were adolescents, according to the World Health Organization criteria [15] (10 to 19 years old) and formed the sample of this study. Each center had a local ethics committee that approved the study. Patients and/or their parents where necessary, signed a written informed consent agreeing with the participation in the study.

The collected data were: current age, age at diagnosis, self-reported color-race (White, Black, Brown (“parda”), Asian (“amarela”) and Indigenous (“indígena”)) [16], diabetes duration, years of school attendance, frequency of self-monitoring of blood glucose (SMBG), smoking status, type of prescribed insulin therapeutic regimens (ITR), self-reported adherence to diet (following at least 80% of the time the prescribed diet) [17] and to prescribed ITRs [14], BMI and self-reported frequency of physical activity (at least three times a week). Family history of diabetes, obesity, hypertension and coronary diseases in first degree relatives were also assessed. The coexistence of another health care insurance, besides that offered by the BNHCS was also assessed.

Adequate glycemic control was defined as the presence of HbA1c levels < 7.5% (58 mmol/mol) [18], and inadequate glycemic control was defined as HbA1c levels  ≥ 7.5% (58 mmol/mol). HbA1c was measured using high-performance liquid chromatography HPLC (Bio-Rad Laboratories, Hercules, California, USA). The last value of HbA1c in the previous year was obtained from the medical records. Fasting triglycerides, HDL cholesterol, total cholesterol and laboratorial markers of NAFLD such as alanine aminotransferase (ALT), aspartate aminotransferase (AST) and gamma-glutamyl transferase, were measured using enzymatic techniques and serum uric acid by an uricase-based commercial (mg/dl). Creatinine was measured using a colorimetric assay kit, corrected for standardized creatinine assay by mass spectrometry. All the above measurements were performed with BioSystem (model A25; Barcelona, Spain). Friedewald’s equation was used to calculate LDL cholesterol values [19]. ITRs were stratified as follows: exclusive use of intermediate insulin (NPH) or regular insulin, long-acting insulin analogs plus short acting insulin or the use of continuous subcutaneous insulin infusion (CSII). Normal weight was defined as a BMI of percentile ≥ 3 and ≤ 85, underweight as a BMI of percentile < 3, overweight as a BMI of > 85th percentile, and obesity as a BMI of > 97th percentile according to age and gender [20] and current smoking as the use of more than one cigarette per day. Elevated blood pressure and hypertension for adolescents were defined according to age, sex and height. Elevated blood pressure was considered as blood pressure values ≥ 90th percentile to < 95th percentile or 120/80 mm Hg to < 95th percentile for children aged 10 to < 13 years, and 120/ < 80 to 129/ < 80 mm Hg for those ≥ 13 years old [21]. Hypertension was defined as systolic blood pressure and/or diastolic blood pressure ≥ 95^th^ percentile or 130/80 to 139/89 mm Hg for children aged 10 to < 13 years and ≥ 130/80 for those with ≥ 13 years old [18, 21]**.** Undesirable levels of LDL-Cholesterol were considered as ≥ 100 mg/dl [18].

#### Sample calculation and economic status evaluation

Sample calculation of the study has been previously described [14, 22]. The sample represented the distribution of T1D cases across four geographic regions of Brazil, estimated using the overall population distribution reported in the 2000 Brazilian Institute of Geography and Statistics (IBGE) Population Census [23], combined with national estimates of diabetes prevalence, to determine the minimum number of patients to be studied in each region [24]. Economic status was defined according to the Brazilian Economic Classification Criteria that takes in account the education level [25]. The following economic status categories were considered: high, middle, low, and very low.

### Diabetes-related chronic complications assessment

#### Evaluation of renal function

Renal function was estimated by the CKD-EPI equation [26] in patients with age ≥ 16 years, by the Schwartz formula in patients younger than 16 years [27] and was expressed as estimated glomerular filtration rate (eGFR) in milliliters per minute per 1.73 m^2^ (mL/min/1.73 m^2^). Albuminuria concentration (immunoturbidimetry, detection limit: 0.01 mg/dl) was measured at least twice from a morning urine sample. The presence of albuminuria was defined as an albuminuria ≥ 30 mg/dl. Patients with normal renal function had an eGFR ≥ 60 mL/min/1.73 m^2^ and the absence of albuminuria. CKD was defined as an eGFR < 60 mL/min/1.73 m^2^, with or without the presence of albuminuria or an eGFR ≥ 60 mL/min/1.73 m^2^ with the presence of albuminuria [28, 29].

#### Evaluation of retinopathy

The screening for diabetic retinopathy (DR) was performed by mydriatic binocular indirect ophthalmoscopy (BIO), EyeTec (OBI OSF) by a retinal specialist. The classification of DR was assessed in the eye that was the most compromised. Each eye was classified based on whether DR was present. Patients were then classified according to the international classification as: absent, non-proliferative diabetic retinopathy (NPDR), proliferative diabetic retinopathy (PDR) and macular edema [30].

### Metabolic syndrome assessment

The definition of MS was done according to the International Diabetes Federation criteria [31]. Adolescents aged 10 to < 16 years old were classified according to the following criteria: (1) abdominal obesity: waist circumference (WC) ≥ 90th percentile for age and gender; (2) triglycerides ≥ 150 mg/dL (1.7 mmol/L); (3) HDL-c < 40 mg/dL (1.03 mmol/L); (4) elevated BP ≥ 130 × 85 mmHg [22]. As there are no reference values of percentiles on abdominal waist in the Brazilian population, we used the same criterion (≥ 90th percentile) for each age and gender group of our sample. Considering that all participants had T1D, central obesity plus an additional factor was necessary for diagnosing MS [32]. Adolescents aged 16 years or older were classified according to the same criteria adopted for adults as follows: central obesity: WC ≥ 90 cm for South American men or ≥ 80 cm in South American women; triglycerides ≥ 150 mg/dL (1.7 mmol/L) or on drug therapy for elevated triglycerides; HDL < 40 mg/dL (1.03 mmol/L) in men or < 50 mg/dL (1.29 mmol/L) in women or on drug therapy for low HDL; elevated BP ≥ 130 × 85 mmHg or using antihypertensive drugs.

### Statistical analysis

For statistical analysis purpose, normal weight and underweight were grouped together as well as overweight and obesity [33]. An exploratory analysis was initially performed, and the data were presented as mean (± SD) or median, interquartile range [IQR] for continuous variables and percentage for discrete variables. Parametric and non-parametric tests were used for comparison between the groups as indicated. Pearson’s correlation coefficient was calculated when applicable.

We have done backward Wald logistic multivariate analysis with overweight/obesity as a dependent variable (outcome variable), and for the independent variables, those with p < 0.2 in exploratory analysis, or those which presented relevance, mainly related to demographic and social data, such as gender, age, diabetes duration, years of school attendance, self-reported color-race, level of care, family history of hypertension, sBP and dBP, use of anti-hypertensive drugs, insulin dose/kg, proportion of basal/bolus insulin doses, geographic region of the country and GFR. Adjustments for social economic status, self-reported color-race and age at diabetes diagnosis were performed. All analyses were performed using the Package for the Social Sciences SPSS version 17.0 (SPSS, Inc., Chicago, Illinois, USA). Odds ratios with 95% confidence intervals (CIs) were calculated where indicated. A two-sided *p* value less than 0.05 was considered to be significant.

## Results

### Overview of the sociodemographic data of the studied population

**Fig. 1:**
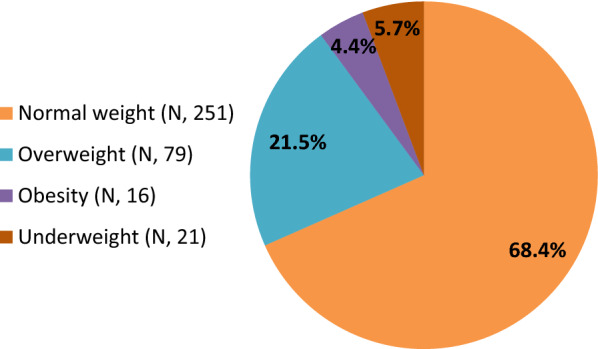
Prevalence of nutritional status of the studied population stratified according to BMI

Overall, 251 patients (68.4%) had normal weight, 21 (5.7%) were underweight and 95 (25.9%) presented overweight/obesity, with 79 patients (21.5%) presenting overweight and 16 (4.4%) obesity (Fig. 1). The sociodemographic data of the studied population are listed in Table 1.

**Table 1:** Clinical and demographic data of the studied population

Variable	
N	367
Age, y	16.4 ± 1.9
Gender, female, n (%)	184 (50.1)
Age at diabetes diagnosis, y	8.9 ± 4.3
Diabetes duration, y	8.1 ± 4.3
HbA1c (%)	9.6 ± 2.4
Ethnicity, n (%)	
Caucasians	176 (48.0)
Geographic region, n (%)	
Southeast	160 (43.6)
North/northeast	133 (36.2)
South	24 (6.5)
Mid-west	50 (13.6)
Economic status	
High	8 (2.2)
Medium	151 (41.1)
Low	193 (52.6)
Very low	15 (4.1)
Level of care n (%)	
Secondary	167 (45.5)
Tertiary	200 (54.5)
Time of follow-up, y	5.4 ± 3.8
Health care insurance (%)	
Public exclusively	263 (71.7)
Public and private	104 (28.3)

*Y* year; data are presented as number (percentage), mean ± SD;

^*^African-Brazilians, Mulattos, Asians, and Native Indians

### Overview of the studied population according to the presence of overweight/obesity

Clinical, demographic and laboratory data stratified according to the presence of overweight/obesity are described in Table 2. Overall, patients with overweight/obesity were female, older, had longer diabetes duration, had more frequently acanthosis nigricans, were attended at a tertiary care level center and used lower insulin doses (U/kg/day) but higher total daily insulin dose (U/day) than those patients without overweight/obesity. No difference was noted concerning the level of HbA1c in the year of the evaluation as well in the previous year. A strong correlation was noted between the last values of HbA1c in the previous year with HbA1c values measured during the study (r = 0.74, p < 0.001). A weak correlation between HbA1c values measured during the study with the levels of total cholesterol (r = 0.244, p < 0.001), triglycerides (r = 0.265, p < 0.001), ALT (r = 0.143, p = 0.007) and AST (r = 0.113, p < 0.035) was noted. No correlation was found with LDL-C and HDL-C values.

**Table 2:** Clinical, demographic and laboratory data stratified by the presence of overweight/obesity

	Overweight/obesity		
	No	Yes	*p-value
N (%)	272 (74.1)	95 (25.9)	
Demographic data			
Gender, female n (%)	127(46.7)	57 (60.0)	0.03
Age, y	16.3 ± 1.9	16.9 ± 1.8	0.005
Diabetes duration, y	7.8 ± 4.2	8.9 ± 4.4	0.04
Age at diagnosis, y	8.8 ± 3.9	8.3 ± 4.2	0.3
Time of follow up, y	5.0[6.0]	5.0 [5.7]	0.7
Level of care, tertiary n (%)	137(50.4)	63(66.3)	0.008
Health insurance(public and private), yes n(%)	80 (29.4)	24(25.3)	0.5
Years of study, y	10.9 ± 2.5	10.8 ± 2.3	0.8
Smoker, yes n(%)	9(3.3)	8(8.4)	0.05
Ethnicity, y (%)†			
Caucasians	127 (46.7)	49 (51.6)	0.4
Geographic region, n (%)			< 0.001
Southeast	98 (36.0)	56 (58.9)	
South	17(6.3)	6(6.3)	
North/Northeast	106(39.0)	28(29.5)	
Mid-west	51(10.9)	5(5.3)	
Economic status (%)			0.5
High	7(2.6)	1 (1.1)	
Medium	111(40.8)	40(42.1)	
Low	141(51.8)	52(54.7)	
Very low	13(4.8)	2(2.1)	
Diabetes management and treatment			
HbA1c (%)	9.7 ± 2.5	9.5 ± 2.3	0.6
HbA1c (mmol/mol)	82.4 ± 26.9	80.9 ± 25.7	
HbA1c < 7.5% n (%)	48(17.7)	16(16.8)	0.6
HbA1c (%) year before HbA1c (mmol/mol), year before	9.8 ± 2.7 84.4 ± 29.8	9.5 ± 2.7 80.7 ± 29.7	0.3
Insulin dose (U/kg/day)	1.05 ± 0.4	0.95 ± 0.4	0.04
Insulin dose, total (U/day)	58.26 ± 21.2	65.67 ± 25.2	0.01
SMBG, yes n (%)	266(97.8)	91 (95.8)	0.3
SMBG, n	3.7 ± 1.4	3.9 ± 1.3	0.18
Adherence to diet, yes n(%)	124 (51.0)	37(44.6)	0.3
Physical activity, yes n(%)^††^	175(64.3)	61(64.2)	0.9
Number of clinical visits/year	3.8 ± 1.7	3.7 ± 1.7	0.95
Diabetes treatment, n(%) **			0.9
NPH or NPH + regular	255 (93.8)	90(94.7)	
Insulin analogs (long or short acting) or CSII	17 (6.2)	5 (5.2)	
Adherence to ITR, yes n(%)	23(13.7)	8(13.6)	0.9
Clinical data			
BMI, kg/m^2^	20,6 ± 2.2	26,7 ± 2.5	< 0.001
Waist circumference, cm	73.6 ± 6.8	87.5 ± 8.5	< 0.001
Systolic blood pressure	111.4 ± 11.4	118.0 ± 10.3	< 0.001
Diastolic blood pressure	68.5 ± 9.0	73.9 ± 8.4	< 0.001
Hypertension, yes n(%)	28 (10.3)	19 (20.2)	< 0.001
Acanthosis yes n(%)	3(1.1)	8(8.4)	< 0.001
Metabolic syndrome, yes n(%)	8(2.9)	30 (31.9)	< 0.001
Laboratorial data			
Uric acid (mg/dL)	4.8 ± 1.4	4.8 ± 1.6	0.6
Total Cholesterol (mg/dL)	182.4 ± 55.2	197.1 ± 57.2	0.03
Triglycerides (mg/dL)	85[58.0]	80.5 [65.7]	0.2
High triglycerides, yes n(%)	40(15.2)	12(13)	0.7
HDL-cholesterol (mg/dL)	54.4 ± 15.4	55.7 ± 18.9	0.7
Low HDL-Cholesterol, yes n(%)	62(23.6)	26(28.3)	0.4
LDL-cholesterol (mg/dL)	106.3 ± 41.8	120.2 ± 39.4	0.006
LDL-cholesterol ≥ 100 mg/dl,n(%)	128(48.5)	57(64)	0.014
Non-HDL-cholesterol(mg/dL)	127.9 ± 50.9	141.3 ± 51.3	0.03
ALT, U/L	13 [8.0]	11[10.0]	0.45
AST, U/L	16[11]	16[12.5]	0.4
GGT, mg/dL	16[10]	18[12]	0.1
Medications			
Metformin, yes n(%)	10 (3.7)	25(26.3)	< 0.001
Anti-hypertensive drugs, yes n(%)	17(6.3)	15(16.0)	0.004
Statins yes n(%)	10(3.7)	9(9.5)	0.03
Family history			
Overweight/obesity, yes n(%)	60(22.1)	20(21.1)	0.8
Type 2 diabetes, yes n(%)	30(11.0)	13(13.7)	0.4
Hypertension	98(36.6)	49(53.3)	0.007
Coronary disease	13(4.9)	6(6.5)	0.5
Diabetes-related chronic complications			
Retinopathy, yes n (%)	19(7.1)	9 (9.7)	0.5
CKD, yes n (%)	33(16.8)	13(16.7)	0.9
GFR, mL/min/1.73m^2^ ***	115.9 ± 32.6	106.8 ± 23.4	0.01
Albuminuria, mg/dL	8.8[13.11]	7.5[16.76]	0.4

The data are presented as n (%), mean ± SD or median [IQR, interquartile range];

†African-Brazilians, Mulattos, Asians, Native Amerindians were considered as non-Caucasians; * p < 0.05 was considered significant. ** For this analysis we considered patients using exclusively insulin provided by the government, free of charge (NPH or Regular) and those using only insulin analogs (long/short acting or CSII), *CSII*  continuous subcutaneous insulin infusion, *ITR* insulin therapeutic regimens, ^††^Physical activity, at least 3/ times per week. *ALT* alanine aminotransferase, *AST* aspartate aminotransferase, *GGT* gamma-glutamyl transferase, *** glomerular filtration rate

The presence of hypertension, family history of hypertension in first degree relatives and MS were higher in patients with overweight/obesityin comparison to patients without this condition. These patients also had higher levels of total and LDL-cholesterol, high frequency of undesirable levels of LDL-Cholesterol, lower level of GFR and were more frequently under the use of metformin, statins and anti-hypertensive drugs. No difference was observed in the levels of ALT, AST, GGT, uric acid, HDL-cholesterol and triglycerides. A similar prevalence of retinopathy and CKD was observed in patients with and without overweight/obesity. Data described in Table 2

### Multivariate logistic analysis with the presence of overweight/obesity as dependent variable

Multivariate analysis performed with the presence of overweight/obesity as a dependent variable, showed that all the independent variables which entered in the model, could explain 20.7% (Nagelkerke R-squared) of a given patient having overweight/obesity. 73.8% of the patients were correctly classified by the model. The presence of overweight/obesity was associated with female gender, age, sBP, and showed a tendency to be associated with geographic region of the country and with the use of anti-hypertensive drugs. Data described in Table 3.

**Table 3:** Final model of logistic regression with overweight/obesity as dependent variable

Variable	B	OR	95% confidence interval	p value
Age, years	0.158	0.171	1.000–1.143	0.05
Gender, female	0.944	2.570	1.454–4.542	0.01
sBP	0.043	1.044	1.010–1.080	< 0.001
Use of anti-hypertensive drugs	− 0.743	0.476	0.214–1.054	0.06
Geographic regions				0.05
Mid-West		1		Reference
Southeast	1.429	4.176	1.362–12.807	0.01
South	0.917	2.502	0.026–2.342	0.213
Northeast/North	0.975	2.651	0.849–8.278	0.09

Adjusted for age at diabetes diagnosis, self-reported color-race and socioeconomic status

*sBP* systolic blood pressure

## Discussion

Our study has shown that almost one quarter of our adolescents with T1D, presented overweight/obesity. Having overweight/obesity was associated with some traditional risk factors for DRCC and CVD such as diabetes duration, hypertension, LDL-cholesterol and MS. The above-mentioned data pointed out that these patients aggregated factors associated with micro and macrovascular DRCC which could translate into poor clinical prognosis in the future [34–36]. Although no association was found with glycemic control (current and in the previous year) it is important to emphasize that less than 20% of the patients in both groups presented an adequate glycemic control. No association was found between overweight/obesity with diabetic CKD, retinopathy and laboratorial markers of NAFLD.

The prevalence of overweight/obesity in patients with T1D ranged from 12 to 38.5% in studies conducted in different countries [2, 14, 37–40]. Our study showed a prevalence of overweight/obesity of about 25% that was within the above-mentioned range for T1D, with no relationship with self-reported color-race and economic status, unlike in the USA, where the prevalence of overweight/obesity was higher among minorities [39]. In multivariate analysis only gender, age, sBP persisted associated with overweight/obesity possibly due to our sample size that was smaller than those evaluated in other studies [13, 37, 38]. Gender, age and sBP have been described as being associated with overweight/obesity in many different studies [13, 14, 39].

A higher prevalence of MS was noted in patients with overweight/obesity in comparison to patients without this clinical condition. It is noteworthy that these latter patients still have a higher prevalence of MS than adolescents without T1D in Brazil which was 1.6% when the IDF criteria were used [41]. A relationship between micro and macrovascular complications in patients with T1D with MS and each of its components has been observed [4, 42]. In our study, hypertension, high sBP and dBP were some of the most important components of MS observed in patients with T1D and overweight/obesity similar to other studies, in T1D [36] and in individuals without T1D [41]. Nevertheless, no association between overweight/obesity with diabetic CKD and retinopathy was observed in the present study. The presence of hypertension, high sBP in the life-course of these patients is a risk factor for the development of these microvascular complications as has been previously demonstrated [43, 44]. Another factor that could also be a background risk factor for the above-mentioned conditions was the family history of hypertension that was higher in the group of patients with overweight/obesity. No difference in the average value of HDL-cholesterol and triglycerides was found in our patients which was not observed in other studies [36, 37]. This could be related to our sample size as well as to demographic characteristics of our population such as lower age and diabetes duration. Similar to other studies performed in patients with T1D, our patients showed a positive correlation between HbA1c and total cholesterol [43]. Patients with overweight/obesity presented higher levels of total and LDL-cholesterol, which result in a higher risk for CVD [37, 45]. We did not measure obesity-related hormones such as ghrelin which could have added some information regarding the pathogenesis of overweight/obesity in this group of patients. However, the role of this hormone in patients with T1D T1D is still controversial [46].

Our data did not show an association between overweight/obesity with the levels of HbA1c, and with the number of patients that reached the targets for good glycemic control. Controversial results have been described in patients with T1D with overweight/obesity concerning glycemic control [7, 12–14, 37]. A Dutch study showed average higher HbA1c levels in patients with overweight/obesity but without difference in the number of patients that presented HbA1c levels < 7.5% [36]. The Finn Dianne study, that included adult patients with MS, showed its association with an inadequate glycemic control [7].

Some studies showed an association between good glycemic control and overweight/obesity probably related to insulin intensive treatment [11, 12]. This fact was not observed in our study. Although the majority of our patients were under the use of multiple insulin injections, less than 50% reported adherence to diet, and also less than 20% reported adherence to IRTs which has an impact upon glycemic control [14, 17]. However, studies that have focused on the levels of HbA1c in patients with T1D, with and without overweight/obesity, have found a difference in HbA1c levels no greater than 0.5%.

An interesting point observed in the present study was the use of lower insulin doses (U/ kg/day) but higher total insulin dose (U/day) in patients with overweight/obesity. This probably occurred because these patients had higher body surface which could be related to insulin resistance that is usually found among these patients [3, 4]. However, it is important to emphasize that the majority of the studies present their data taking in account insulin dosage as U/Kg/day, and the results regarding the presence of overweight/obesity is still controversial [4, 33]

Finally, the use of metformin had a negative effect on overweight/obesity. It was used as adjunct therapy to insulin by 26.3% of our patients with overweight/obesity, mainly females (data not shown). Although these patients had similar levels of HbA1c, they used lower insulin doses than those with normal weight. Other studies that have also evaluated metformin in overweight/obese patients with T1D showed that metformin use was associated with significant reductions in HbA1c levels and insulin doses, with no significant changes in weight [47]. Another study, conducted in Denmark, using metformin or placebo as adjunct to insulin did not find a significant difference on HbA1c levels, but the insulin doses and weight showed significant reductions [48]. As expected, patients with overweight/obesity were more frequently using anti-hypertensive drugs and statins.

Our data were obtained and evaluated very uniformly and had a broad spectrum, which led us to evaluate many covariates, which was, consequently, a strength of our study.

Some limitations of our study must also be mentioned. Firstly, similar to other epidemiologic studies conducted with T1D [13, 33], we did not measure C peptide levels, nor autoantibodies against beta cells, and used only clinical criteria for T1D diagnosis. Secondly, all the information about adherence to diet and to ITRs as well as practice of regular physical activity were self-reported. Thirdly, as we did not have the weight of the patients at the moment the diagnosis was made, we could not know if the presence of overweight/obesity was already present since then. Finally, as our study had a cross-sectional design, a causal relationship between those factors found to be associated with overweight/obesity and the presence of this clinical condition cannot be established.

## Conclusions

Almost one quarter of our patients presented overweight/obesity. These patients had higher prevalence of traditional risk factors for micro and macrovascular DRCC such as diabetes duration, hypertension, high levels of LDL-cholesterol and presence of MS. The majority of patients with or without overweight/obesity presented inadequate glycemic control that is also an important risk factor for these complications. No association was found between overweight/obesity with diabetic CKD, DR and laboratorial markers of NAFLD. The above-mentioned data point out that further prospective studies are urgently needed to establish the clinical prognosis of these young patients.

## Data Availability

The datasets used and/or analyzed during the current study are available from the first author on reasonable request.

## Abbreviations

T1D: Type 1 diabetes
MS: Metabolic syndrome
DCCT: The Diabetes Control and Complications Trial
EDIC: The Epidemiology of Diabetes Interventions and Complications
BMI: Body mass index
HbA_1_C: Glycated hemoglobin
NPH: Neutral protamine Hagedorn
BNHCS: Brazilian National Health Care System
SMBG: Self-monitoring of blood glucose
ITR: Insulin therapeutic regimen
HPLC: High-performance liquid chromatography
ALT: Alanine aminotransferase
AST: Aspartate aminotransferase
CSII: Continuous subcutaneous insulin infusion
sBP: Systolic blood pressure
dBP: Diastolic blood pressure
IBGE: Brazilian Institute of Geography and Statistics
CKD: Chronic kidney disease
eGFR: Estimated glomerular filtration rate
DR: Diabetic retinopathy
NPDR: Non-proliferative diabetic retinopathy
PDR: Proliferative diabetic retinopathy (PDR)
WC: Waist circumference
DRCC: Diabetes-related chronic complications
CVD: Cardiovascular diseases
